# Summer Diarrhœa and Cholera

**Published:** 1889-07-06

**Authors:** William Moore

**Affiliations:** K.C.I.E., late Surgeon-General with the Government of Bombay


### Summer Diarrhœa and Cholera.

By Sir William Moore, K.C.I.E., late Surgeon-General with the Government of Bombay.

In the summer time, and especially when the season is a hot one, diarrhœa begins to prevail, and often spreads, particularly among children, with epidemic intensity, causing a considerable increase in the death-rate. Yet, although this is the case, the term diarrhœa is not to the general public of the same grave import as the term cholera. It may therefore surprise many persons if they are told that there is every reason for asserting that English summer diarrhœa is in reality a minor phase of cholera. There is an impression abroad that cholera is a disease always characterised by certain distinctive symptoms of which cramps, suppression of urine, and evacuations like water in which rice has been boiled, are the principal. No doubt a typical case of cholera is so illustrated, but very many cases are not typical. Whenever a cholera epidemic occurs, there are as many cases of diarrhœa as of cholera, and most of the cases of cholera commence as diarrhœa. When the latter malady presents itself during an epidemic of cholera, no one can possibly foretell what the result may be. Yet the malady is not called cholera until certain additional symptoms appear. This is not consistent with what is admitted with regard to other diseases. No one would hesitate to diagnose a case as diphtheria, or scarlet fever, or enteric fever, even although all the typical symptoms were not present.

There are few maladies which present under more varied aspects than cholera. Several authors have remarked that every cholera epidemic presents a peculiar train of symptoms. Several epidemics have occurred when the evacuations, instead of being like rice water, were tinged with blood (*cholera hæmorrhagica*). In other epidemics the malady has been so like remittent fever that the question has presented, which of the diseases were to be treated. In other epidemics the majority of the cases have afforded symptoms like those of the sweating sickness of former centuries. But the variability of individual cases is still more pronounced. Rice-water evacuations may be absent, especially in the old; black vomits may occur, as in yellow fever; death may occur at once from collapse, without vomiting or purging; cramps have been noted without other symptoms; increased flow of urine has also been recorded; all the symptoms of cholera may occur, excepting purging: diarrhœa may be the principal feature.

It was remarked above that cases commencing as diarrhœa frequently terminate as cholera. But there are other instances which from their commencement are so like cholera that they would be unhesitatingly recorded as cholera, if cholera were in the neighbourhood. In fact, the only difference is that the symptoms in bad cases of summer diarrhœa are a little less severe than those of Asiatic cholera. But degree is not sufficient to warrant the diseases being essentially different in their nature. There are mild and severe types of most maladies, and cholera is not an exception.

If we refer to the presumed causes of cholera, we find that cholera has been ascribed to a microbe or germ having its habitat in the upper layers of the ground, requiring, according to Pettenkofen, a certain degree of porosity of the ground and a certain degree of moisture. Very recently Dr. Ballard has (provisionally) referred summer diarrhœa to a connection with a micro-organism in the upper layers of the earth, which is apparently most active when a thermometer shows a temperature of 56deg. at four feet deep from the surface, no matter what the external atmospheric heat may be. And it is also surmised that porosity of ground is essential, as diarrhœa prevails most on earth having such character.

But this is not all. The pathology of summer diarrhœa assimilates greatly to that of cholera; the sequelæ of the two conditions may be the same, especially in the occurrence of a typhoid febrile condition. Cholera prevails most in hot climates, and diarrhœa prevails most in the hottest weather of temperate climates, and the best treatment for bad summer diarrhœa is the best treatment for cholera.

Whether we consider the diversity of the symptoms of cholera, or the manner in which it commences, or the climates in which cholera and summer diarrhœa prevail, or their epidemic spread, or the resemblance of the symptoms of summer diarrhœa to cholera, or the sequelæ of the two maladies, or the pathology, or the presumed causes, or the best treatment, the conclusion that summer diarrhœa and cholera are one and the same disease appears inevitable.

Experience has taught me that cholera is not always to be diagnosed by the distinctive symptoms found in text-books. Experience has taught me that cholera must be met in various insidious forms, and one of these insidious phases is summer diarrhœa.

But the recognition of the analogy of summer diarrhœa and cholera should not create uneasiness or scare. This is unreasonable, for even in the worst epidemics of cholera, immunity is the rule. And summer diarrhœa will not be any more severe than it has hitherto been if recognised as what it really is—viz., a milder phase of cholera. On the other hand, such recognition is calculated to further that sanitary vigilance by which both phases of the malady may be prevented.

